# Sex differences in chest pain presentation, triage assessment, and outcomes in urgent primary care: findings from the TRACE cohort study

**DOI:** 10.1017/S1463423625100182

**Published:** 2025-07-02

**Authors:** Amy Manten, Bryn Hummel, Renee Bolijn, Remco P. Rietveld, Irene G.M. van Valkengoed, Eric P. Moll van Charante, Ralf E. Harskamp

**Affiliations:** 1 Amsterdam UMC, University of Amsterdam, Academic Medical Center, Department of General Practice, Meibergdreef 9, 1105 AZ Amsterdam, The Netherlands; 2 Amsterdam Cardiovascular Sciences, Atherosclerosis & Ischemic syndromes, Amsterdam, The Netherlands; 3 Amsterdam UMC, University of Amsterdam, Academic Medical Center, Department of Public and Occupational Health, Amsterdam Public Health, Amsterdam, The Netherlands; 4 Huisartsenorganisatie Noord-Kennemerland, Hertog Aalbrechtweg 5A, 1823 DL Alkmaar, The Netherlands

**Keywords:** Acute coronary syndrome, chest pain, primary care, sex differences, triage

## Abstract

**Aim:**

To evaluate sex differences in the triage and assessment of chest pain in Dutch out-of-hours primary care (OOH-PC).

**Background:**

Prior research illustrated differences between women and men with confirmed cardiac ischemia. However, information on sex differences among patients with undifferentiated chest pain is limited and current protocols used to assess chest pain in urgent primary care in the Netherlands do not account for potential sex differences.

**Methods:**

A retrospective cohort study of consecutive patients who contacted a large OOH-PC facility in the Netherlands in 2017 regarding chest pain. We performed descriptive analyses on sex differences in patient and symptom characteristics, triage assessment, and subsequent clinical outcomes, including acute coronary syndrome (ACS).

**Findings:**

A total of 1,802 patients were included, the median age was 54 years, and 57.6% were female. Compared to men, women less often had a history of cardiovascular disease (CVD) (16.0% vs 25.8%, p < 0.001) or cardiovascular risk factors (49.3% vs 56.0%, p = 0.005). Symptom characteristics were comparable between sexes. While triage urgencies were more frequently altered in women, the resulting triage urgencies were comparable, including ambulance activation rates (31.1% and 33.5%, respectively, p = 0.33). Musculoskeletal causes were the most common in both sexes; but women were less likely to have an underlying cardiovascular condition (21.1% vs 29.6%, p < 0.001), including ACS (5.4% vs 8.5%, p = 0.019).

**Conclusion:**

Women more frequently sought urgent primary care for chest pain than men. Despite a lower overall risk for cardiovascular events in women, triage assessment and ambulance activation rates were similar to those in men, indicating a potentially less efficient and overly conservative triage approach for women.

## Introduction

Chest pain is a common medical complaint in primary care. During office hours, 0.7%–2.7% of all general practitioner (GP) consultations are related to chest pain, of which the majority usually concern female patients (Bösner *et al.*, 2009, Frese *et al.*, 2016, Nilsson *et al.*, 2003, Svavarsdóttir *et al.*, 1996, Verdon *et al.*, 2008). In out-of-hours primary care (OOH-PC) settings, chest pain is among the top 10 of chief complaints (Jansen *et al.*, 2020). Chest pain often presents a diagnostic dilemma, as the majority of patients suffer from non-life-threatening conditions, while severe underlying cardiovascular conditions, including acute coronary syndrome (ACS), are only present in approximately one out of every 8–12 patients (Hoorweg *et al.*, 2017, Nilsson *et al.*, 2003, Wouters *et al.*, 2020).

To aid adequate patient assessment in OOH-PC, triage protocols are in place, which rely on a number of patient and symptom characteristics. Remarkably, the patient’s sex is not included in the Dutch triage protocols. This is relevant since prior studies on selected populations with proven cardiac ischemia showed more atypical symptom presentations, increased delay in diagnosis and treatment, and subsequent poorer outcomes in women (Arora and Bittner, 2015, Chiha *et al.*, 2015, Dawson *et al.*, 2023, Jones *et al.*, 2012, Mehilli and Presbitero, 2020, Mehta *et al.*, 2016, Perdoncin and Duvernoy, 2017, Shaw *et al.*, 2006, Shin *et al.*, 2010). However, information on sex-related differences in triage assessment of patients with undifferentiated chest pain is limited, and the applicability of the triage protocols in women and men separately is insufficiently examined.

Considering the broad range of possible underlying causes of chest pain and the importance of safe and efficient risk stratification in both women and men presenting with chest pain, we aimed to assess sex differences throughout the current Dutch triage protocol for chest pain in urgent primary care. We evaluated differences in patient- and symptom characteristics, triage assessment, and subsequent clinical outcomes in adult women and men who contacted an OOH-PC facility with a chief complaint of chest pain.

## Methods

We reported our study in accordance with the Strengthening the Reporting of Observational Studies in Epidemiology (STROBE) guideline (von Elm *et al.*, 2007). The study protocol was approved by our institution’s Medical Ethical Committee and registered in the Dutch Trial Register (TRACE NL7581) (Register, 2011).

This analysis is part of the ‘TRiage of Acute Chest pain Evaluation in primary care’ (TRACE) cohort study (Manten *et al.*, 2020). In short, we performed a retrospective, observational cohort study of patients who contacted the OOH-PC facility in Alkmaar, the Netherlands, in 2017, with chest pain as the main reason for contact. At the outset of the study, all patients received information by mail and were provided with the opportunity to opt-out from sharing data. A more elaborate description of TRACE study methods was published previously (Manten *et al.*, 2020) and can also be found at https://www.amstelheart.nl.

### Background and healthcare setting

In the Netherlands, all citizens are registered with a local GP’s office that holds an electronic patient record file. When offices are closed, urgent primary care is handled through regional OOH-PC facilities (Smits *et al.*, 2017). The facility in Alkmaar is responsible for OOH-PC services for 98 affiliated GPs, representing a community of approximately 240,000 individuals living in both urban and rural areas.

To handle the high demand for care, OOH-PC facilities operate a (telephone) triage system, executed by trained triage assistants following standardised computer-based protocols by the Netherlands Triage Standard (NTS) (Domus Medica, 2014). After a short check of the patient’s hemodynamic status (mainly by assessing consciousness and breathing), the protocol for chest pain continues with 7 hierarchically structured questions regarding the characteristics of the complaint (i.e. type, duration, severity, course, location, pain radiance, and associated symptoms) (Domus Medica, 2014, Zeilstra and Giesen, 2017). Based on the answers, the NTS algorithm calculates an urgency category, varying from urgency level 1 to 5, linked to a recommended ‘time-until-care’ (i.e. U1 – most urgent, immediate care, U5 – non-urgent, care within 24 hours). Triage assistants include their own assessment of the patient’s condition and may overrule the proposed urgency code after consulting the attending physician. Possible responses following telephone triage include ambulance activation, consultation by a GP (either at the OOH-PC facility or at the patient’s home), telephone advice, and deferral to the own GP during office hours.

### Study population and size

**Figure 1.**
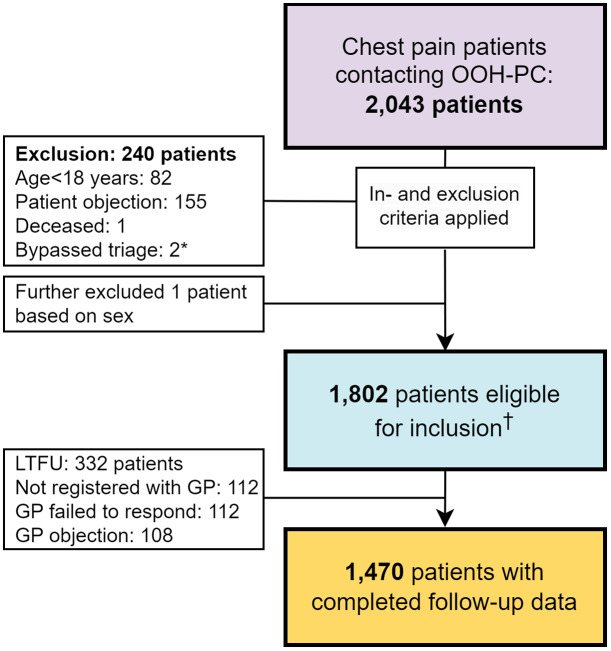
Flowchart showing in- and exclusion of patients. A total of 2,043 patients contacted the OOH-PC facility regarding chest pain in 2017. During exclusions, one additional participant had to be excluded from current analyses due to unclear information regarding their sex. *Two patients bypassed the triage system entirely and were excluded from the dataset. ^†^Among the 1,802 included patients, 23 patients were deemed hemodynamically unstable, resulting in discontinuation of triage and immediate action. Symptom characteristics were therefore analyzed among the remaining 1,779 patients. Follow-up data including final diagnoses were gathered in 1,470 patients. Abbreviations: out-of-hours primary care (OOH-PC), loss to follow-up (LTFU), general practitioner (GP).

We enrolled consecutive women and men contacting the OOH-PC facility with chest pain between January 1^st^, 2017, and December 31^st^, 2017. We excluded patients who objected to participate in the study, patients aged <18 years, patients who bypassed the triage process, and patients who had already died and for whom the OOH-PC was requested for a visit for formal death confirmation (Figure 1). Patients who directly contacted emergency services (i.e. ambulance services) were not enrolled.

### Data collection and outcomes of interest

Data from the digital triage registry were linked to information from the GP’s patient record file. After de-identification, data were entered into a secure electronic data capturing platform (Castor EDC, Ciwit BV, Amsterdam, The Netherlands) (Ciwit, 2016). Gathered data include patient demographics (i.e. age and sex, defined as either male or female), medical history (with specific attention for cardiovascular risk factors and sex-specific risk factors), triage results, and clinical outcomes. We aimed our main focus on cardiovascular outcomes (including ACS). Secondarily, we assessed the occurrence of a major event within 6 weeks of initial contact. We defined major events as a composite of all-cause mortality, and both cardiovascular and non-cardiovascular urgent conditions, that were linked to the initial complaint of chest pain, and required hospital admittance or urgent in-hospital treatment (*supplement 1*) (Manten *et al.*, 2022). Final diagnoses were verified using source documentation, including hospitalisation and/or discharge letters related to the index consultation. We countered inter-observer variability between investigators by the use of standard operating procedures and by an internal audit of patients’ baseline data.

### Data analysis

Sex differences in patient- and symptom characteristics, triage results, and clinical outcomes were assessed using the Mann–Whitney-U test (continuous variables) and chi-square tests (categorical variables). P-values <0.05 were considered statistically significant. Within triage results, we focused on urgency code adjustments made by the triage assistants. We performed logistic regression modelling to identify factors associated with urgency code alterations. We included sex, age, variables indicating cardiovascular burden (i.e. prior cardiovascular disease (CVD) and cardiovascular risk factors), and symptom characteristics suggestive of an ACS (i.e. tightness, severe pain, presence of pain radiance, and associated symptoms). Furthermore, we conducted subgroup analyses comparing women and men diagnosed with ACS to identify possible sex differences among ACS patients. We assessed the diagnostic accuracy of U1 urgency allocation for women and men with ACS using sensitivity and specificity.

Categorical data were reported as frequencies. Non-normal distributed continuous variables were identified using histograms and expressed as medians (IQR). All analyses were carried out using IBM SPSS Statistics for Windows, version 28 (IBM Corp., Armonk, N.Y., USA).

## Results

### Patient characteristics

Our study population consisted of 1,802 patients, of which 1,038 (57.6%) were female (Figure 1). The median age was 54 years in both sexes (Table 1). Female patients less frequently had a history of CVD or cardiovascular risk factors compared to male patients. Out of the total study population, immediate ambulance activation was followed in 23 patients with signs of hemodynamic instability, this number was comparable between women and men (n = 17 vs n = 6, p = 0.14). In the remaining 1,779 patients (57.4% women), triage was followed with detailed assessment of symptom characteristics. While we found no sex differences in most symptom characteristics, we observed subtle differences, as men more often reported mild pain and left-sided localisation compared to women, while women more often reported retrosternal pain and radiance (both typical and atypical).

**Table 1.** Patient and symptom characteristics among the women and men in our study population

	Women	Men	p
**Patient characteristics**	1,038 (57.6)	764 (42.4)	<0.001
Age	54 (36–70)	54 (37–70)	1.00
Prior CVD^*^	166 (16.0)	197 (25.8)	<0.001
Cardiovascular risk factors^†^	512 (49.3)	428 (56.0)	0.005
**Symptom characteristics**	1,021 (57.4)	758 (42.6)	<0.001
*Type of pain*			
Tightness/pressure	503 (49.3)	344 (45.4)	0.11
Stabbing/sharp	240 (23.5)	197 (26.0)	0.23
Fixed to respiration	113 (11.1)	88 (11.6)	0.72
Unknown/unclear	154 (15.1)	116 (15.3)	0.90
*Duration of chest pain*			
<12 hours	604 (59.2)	465 (61.3)	0.35
>12 hours	414 (40.5)	292 (38.5)	0.39
*Chest pain severity (1-10 point scale)*			
Mild (<4)	158 (15.5)	162 (21.4)	0.001
Moderate (5–7)	499 (48.9)	345 (45.5)	0.16
Severe (8–10)	155 (15.2)	107 (14.1)	0.53
Pain has subsided/no pain	190 (18.6)	129 (17.0)	0.39
*Course of chest pain*			
Gradual increase in intensity	504 (49.4)	361 (47.6)	0.47
Atypical and subsided	127 (12.4)	93 (12.3)	0.91
Typical and subsided	29 (2.8)	32 (4.2)	0.11
Rapidly progressive	58 (5.7)	44 (5.8)	0.91
*Location on the chest*			
Left chest	305 (29.9)	268 (35.4)	0.014
Right chest	149 (14.6)	101 (13.3)	0.45
Middle (retrosternal)	329 (32.2)	210 (27.7)	0.040
*Pain radiation* ^‡^			
Typical cardiac radiation	307 (30.1)	189 (24.9)	0.017
Atypical radiation	147 (14.4)	84 (11.1)	0.040
Location not specified	72 (7.1)	59 (7.8)	0.56
No radiation	410 (40.2)	331 (43.7)	0.14
*Associated symptoms* ^§^			
Present	214 (21.0)	157 (20.7)	0.90
Subsided	50 (4.9)	46 (6.1)	0.28
No associated symptoms	647 (63.4)	471 (62.1)	0.60

Patient flow in the table: in total, 1,802 patients were included in the study; 1,038 were women and 764 were men. When assessing symptom characteristics, 1,779 patients (1,021 women and 758 men) could be analyzed. In the remaining 23 patients, triage was discontinued before symptoms could be discussed due to signs of hemodynamic instability (e.g. unconsciousness or severe dyspnoea). In these patients, an ambulance was deployed immediately.

* Cardiovascular disease (CVD) was defined as a history of myocardial infarction, transient ischemic attack, cerebrovascular accident, peripheral artery disease, previous percutaneous coronary intervention, and/or coronary artery bypass grafting.

† Cardiovascular risk factors include prior CVD, a history of diabetes, hypertension, hypercholesterolemia, obesity, smoking and/or a positive family history for cardiovascular disease. For women, a history with gestational hypertension or (pre)eclampsia were also considered cardiovascular risk factors.

‡ Pain radiance was considered typical when the arm(s), shoulder(s), jaw(s), or throat were affected. All other locations of radiation were considered atypical.

§ Associated symptoms include the presence of sweating, nausea, vomiting, pallor, anxiety, fainting, and/or near collapse.

Continuous data are presented as a median and interquartile range due to a non-normal distribution. All categorical data are presented as number and percentage.

### Triage: urgency code assignment and resulting action

In both women and men, triage often resulted in the allocation of urgency codes U1-U3, as is shown in Table 2. We found that in female patients, the initial urgency code was more frequently altered compared to men (16.4% vs 12.9%, p = 0.045). After adjusting for possible confounders, female sex remained a factor associated with urgency code alteration (also see Table 3). Alterations can be divided in ‘downscaling’ (58.9% of revised urgencies, in 9.7% of women and 7.5% of men, p = 0.11), ‘upscaling’ (32.1%, in 5.2% of women and 4.2% of men, p = 0.34), and urgency codes that eventually remained equal to initial ones. Alterations of urgency codes by age strata can be found in *supplemental figure 2*.

**Table 2.** Urgency code allocation following triage

	NTS urgency			Final urgency		
	Women (n = 1,021)	Men (n = 758)	**p**	Women (n = 1,021)	Men (n = 758)	**p**
U1	367 (35.9)	278 (36.7)	0.75	295 (28.9)	238 (31.4)	0.25
U2	237 (23.2)	156 (20.6)	0.19	322 (31.5)	194 (25.6)	0.006
U3	335 (32.8)	262 (34.6)	0.44	330 (32.3)	267 (35.2)	0.20
U4	3 (0.3)	2 (0.3)	1.00	3 (0.3)	5 (0.7)	0.30
U5	49 (4.8)	42 (5.5)	0.48	41 (4.0)	35 (4.6)	0.54
Not registered	30 (2.9)	18 (2.4)	0.47	30 (2.9)	19 (2.5)	0.58

The left side of the table illustrates the initial urgency codes calculated by the NTS algorithm based on the registered answers to the triage questions. On the right side of the table, the final urgency codes are displayed. These represent the urgency code allocation after correcting for revisions made by the triage assistants (i.e. up- and down-scaling).

Definition of urgency codes and their recommended ‘time-until-care’.

U1: Life threatening (immediate: ambulance activation).

U2: Emergent (care <60 minutes).

U3: Urgent, circumstantial risk of arm (care within several hours).

U4: Non-urgent, low risk of harm (care within 24 hours).

U5: No risk of harm (advice).

**Table 3.** Results of multivariable logistic regression for urgency code alteration

Variable	Coefficient	Significance	Exp (β)	95% Confidence interval
Age^*^	−0.090	0.023	0.914	0.846 – 0.988
Female sex	−0.274	0.049	0.760	0.578 – 0.999
Prior CVD^†^	−0.197	0.38	0.821	0.531 – 1.270
Cardiovascular risk factors^‡^	0.017	0.92	1.017	0.746 – 1.386
Tightness/pressure type pain	−0.297	0.046	0.743	0.554 – 0.995
Severe pain	−0.064	0.75	0.938	0.630 – 1.398
Typical radiation^§^	−0.036	0.83	0.965	0.690 – 1.349
Associated symptoms present	−0.061	0.75	0.941	0.651 – 1.360

* Age was stratified in age-categories (18–29 | 30–39 | 40–49 | 50–59 | 60–69 |70–79 | 80–89 | >90).

† Cardiovascular disease (CVD) was defined as a history of myocardial infarction, transient ischemic attack, cerebrovascular accident, peripheral artery disease, previous percutaneous coronary intervention, and/or coronary artery bypass grafting.

‡ Cardiovascular risk factors include prior CVD, a history of diabetes, hypertension, hypercholesterolemia, obesity, smoking, and/or a positive family history for cardiovascular disease. For women, a history of gestational hypertension or (pre)eclampsia were also considered cardiovascular risk factors.

§ Radiation was considered typical when the arm(s), shoulder(s), jaw(s), or throat were affected.

The action following triage showed no significant differences between women and men. In both, telephone triage most frequently resulted in GP consultation, either at the OOH-PC facility or through a home visit (women 56.0%, men 55.3%, p = 0.75). Ambulance deployment followed in 31.3% of women and 33.5% of men (p = 0.33).

### Clinical outcomes

Follow-up information, including clinical outcomes, was available for 1,470 (81.6%) patients, with comparable sex ratios to our total sample (57.8% women). A musculoskeletal cause of chest pain was found most commonly in both women and men (Table 4). Major events occurred more often in men compared to women (129 (20.8%) vs 109 (12.8%), p < 0.001). The majority of major events were of cardiac origin, including ACS, atrial fibrillation, or heart failure. *Supplemental Table 3* provides an overview of specific major event diagnoses for women and men. We found no difference in fatal outcomes, with fatal outcomes in 13 (1.5%) women and nine (1.4%) men during the first 6 weeks following index presentation (p = 1.00).

**Table 4.** Distribution of final diagnoses

	Women (n = 849)	Men (n = 621)	p
Cardiovascular diagnosis	179 (21.1)	184 (29.6)	<0.001
Acute coronary syndrome	46 (5.4)	53 (8.5)	0.019
Musculoskeletal	399 (47.0)	274 (44.1)	0.28
Respiratory	75 (8.8)	50 (8.1)	0.60
Abdominal	98 (11.5)	54 (8.7)	0.08
Psychological and mental health	62 (7.3)	41 (6.6)	0.60
Other	36 (4.2)	18 (2.9)	0.18

The table shows the distribution of final diagnoses among women and men with completed follow-up data.

*Abbreviations:* acute coronary syndrome (ACS).

### Triage of patients with ACS

**Figure 2.**
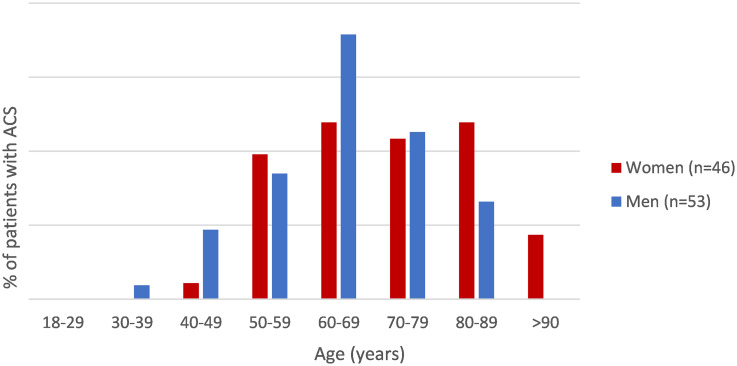
Patients diagnosed with ACS, stratified by age and sex. The figure illustrates the difference between the 46 women and 53 men with an ACS, divided in decimal age groups. P-values: 1.00 (30–39), 0.21 (40–49), 0.74 (50–59), 0.20 (60–69), 0.91 (70–79), 0.17 (80–89), 0.043 (>90).

In total, 99 patients were diagnosed with ACS, 53 (8.5%) men and 46 (5.4%) women (p = 0.019). *Supplemental Table 4* provides a comparison of characteristics between women and men with ACS. Among these selected patients, there were no significant differences in patient- (i.e. age, CVD history, and cardiovascular risk factors) and symptom characteristics. When patients with an ACS are stratified by age and sex (Figure 2), differences are noticeable among patients aged 60-69 years, however not statistically significant (p = 0.20). The difference between women and men aged >90 is significant (p = 0.043). Triage results were also similar among female and male ACS patients. In women, final U1 codes were allocated to 33/46 ACS patients, reflecting a sensitivity of 71.7% [95%-CI: 56.5–84.0] and specificity of 71.2% [68.0–74.3]. In men, final U1 codes were registered in 34/53 ACS patients, with a corresponding sensitivity and specificity of 64.2% [49.8–76.9] and 71.0% [67.0–74.7], respectively. Furthermore, triage resulted in ambulance activation in 82.6% of women with an ACS and 66.0% of men with an ACS, p = 0.06.

## Discussion

In this study, we evaluated possible differences between women and men in patient- and symptom-characteristics, triage assessment, and clinical outcomes of acute chest pain in a regional, out-of-hours urgent primary care facility. We found that women more often contact OOH-PC with chest pain and that women who contacted the facility had an overall lower a priori cardiovascular risk, and similar symptom presentation when compared to men. While urgency code alterations were more common in women, resulting actions were comparable. Women were less likely to have an underlying major event, including cardiovascular diagnoses and ACS. The most commonly reported final diagnosis among both women and men was musculoskeletal pain.

### Strengths and limitations

Our study involved a large consecutive sample of 1,802 patients who underwent structural and protocol-mandated symptom- and risk-assessment at a large-scale OOH-PC facility. Therefore, the results are likely to be generalisable to other OOH-PC facilities in the Netherlands. We optimised data quality by an internal audit of baseline data and by adjudication of registered final diagnoses.

The first and most important limitation is the presence of differential verification bias. Our study involved routine care data from a primary-care-based, retrospective, and observational study, in which we had no influence on the diagnostic work-up following primary care assessment. Therefore, although unlikely, the absence of urgent clinical outcomes (such as missed ACS cases) cannot be guaranteed in patients who were not referred to secondary care. Moreover, in a mentionable number of patients, data on clinical outcomes could not be obtained due to GPs failing to respond, or GPs who expressed liability concerns due to the recent implementation of European privacy regulations. The latter led them to refuse to provide follow-up data for their patients, despite the proven validity of the opt-out procedure (Ploem *et al.*, 2020). However, this did not appear to differently affect female and male patients; hence, the consequences for our stratified analyses may be limited.

### How our findings fit in with existing literature

Overall, the majority of our patients were female, and in both sexes, the majority of final diagnoses were of musculoskeletal origin. Both of these findings are consistent with previous research in acute primary care settings (Bösner *et al.*, 2009, Svavarsdóttir *et al.*, 1996, Verdon *et al.*, 2008). The higher incidence of ACS among men with chest pain compared to women is supported by earlier findings (Arora and Bittner, 2015, Hillinger *et al.*, 2017, Leite *et al.*, 2015, Rubini Gimenez *et al.*, 2014), as well as by two other Dutch studies conducted between 2013 and 2016 that illustrated ACS incidences of around 8% for women and 14% for men (van der Meer *et al.*, 2019, Wouters *et al.*, 2020). Furthermore, in these studies, triage resulted in equally high urgency codes in women and men (i.e. U1 or U2 code in 68.7% of women and 68.2% of men in Wouters et al.; 65.6% and 64.9% in van der Meer et al, respectively). In our study, 60.4% of women and 57.0% of men received either a U1 or U2 code (p = 0.15), and ambulance activation rates were similar as well. Thus, despite a lower a-priori risk of ACS, triage assessment is comparable between women and men, leading to relatively more cautious triage in women (i.e. more unnecessary high urgencies).

This increased cautiousness in pre-hospital care may be related to the rising attention for unrecognised CVD in women. Although contradictory to our own results, others reported differences in the management of women and men with acute chest pain in prehospital settings. An Australian study by Dawson et al. (Dawson *et al.*, 2023) assessed sex differences in the care pathway from emergency medical services (i.e. ambulance services) through to clinical outcomes following discharge. At the outset, women had a less pronounced risk profile compared to men, and throughout the care pathway, women (both total and ACS diagnosed) were less likely to receive guideline-directed care (e.g. more delays, less likely to be admitted or undergo coronary angiography). Eventually, mortality rates were higher among the women with a STEMI compared to men with STEMI. Albeit, many of these outcome measures concern management in secondary care and surpass the urgent primary care setting that we studied.

When we pursue the direction towards secondary care further, we see that research on sex differences among selected, hospital-based patients with cardiac ischemia shows conflicting results. Some studies illustrate that women with ACS are more likely to have an atypical symptom presentation, report chest pain less frequently, and tend to experience more additional symptoms, such as back pain, loss of appetite, and nausea (Arora and Bittner, 2015, Arslanian-Engoren and Engoren, 2010, Shin *et al.*, 2010). In contrast, studies that employ systematic symptom reporting illustrate more similarities than differences, both in primary and secondary care settings (DeVon *et al.*, 2014, Mackay *et al.*, 2011). While speculative, these differences might be the result of reporting and spectrum biases. At the very least, we can state that findings in urgent primary care are consistent in that sex differences are limited, and overall a more cautious triage route is opted in women, which in hindsight leads to more unnecessary ambulance deployments and emergency care evaluation in women than men.

### Implications for further research

Adequate triage of undifferentiated chest pain remains a challenge in both women and men. Our study found that women and men are managed equally by the current triage system. Triage results in similar actions – despite differences in a priori risk of urgent clinical outcomes – resulting in relatively more cautious triage in women compared to men. Based on these results, future efforts to improve triage should investigate whether the addition of sex enhances risk stratification, independently from the cardiovascular risk burden.

Furthermore, a more extensive patient sample, also including patients with chest pain who contacted the national emergency number (i.e. ambulance services) would be beneficial to our understanding of urgent pre-hospital care. In addition, as prior research shows that ACS patients do not exclusively present themselves with chest pain, evaluation of other primary complaints, such as dyspnoea, would be valuable.

## Conclusion

Our study illustrates more similarities than differences between women and men presenting with chest pain to urgent primary care. Women generally exhibit a lower cardiovascular risk profile and experience fewer adverse cardiovascular outcomes, including ACS. Despite this lower risk, current triage protocols do not account for these sex differences, resulting in a less efficient and relatively conservative triage approach for women. Future research should explore the potential benefits of incorporating sex as an additional risk factor in risk stratification tools.

## Supporting information

Manten et al. supplementary material 1 Manten et al. supplementary material

Manten et al. supplementary material 2 Manten et al. supplementary material

Manten et al. supplementary material 3 Manten et al. supplementary material

Manten et al. supplementary material 4 Manten et al. supplementary material

## Supplementary material

The supplementary material for this article can be found at https://doi.org/10.1017/S1463423625100182.
