# Supplementary material for: Sex differences in chest pain presentation, triage assessment, and outcomes in urgent primary care: findings from the TRACE cohort study
Source: Prim Health Care Res Dev. 2025 Jul 2;26:e53. doi: 10.1017/S1463423625100182 (PMC12260727; doi:10.1017/S1463423625100182)
Supplement: Manten et al. supplementary material 1 — Manten et al. supplementary material [file S1463423625100182sup001.docx]

**Supplement 1.** Definition of major and non-major events

|  | **Final diagnosis** | **Condition** |
| --- | --- | --- |
| **Major event** | Death from any cause |  |
|  | Acute coronary syndrome |  |
|  | Urgent coronary revascularization |  |
|  | Pulmonary embolism |  |
|  | Thoracic aortic aneurysm (dissection or ruptured) |  |
|  | Severe/Acute congestive heart failure | Hospitalization |
|  | Severe peri(myo)carditis | Hospitalization |
|  | Symptomatic atrial fibrillation | Hospitalization (cardioversion or converted through medication) |
|  | Aortic valve stenosis | Hospitalization |
|  | (Tension) pneumothorax | Hospitalization |
|  | Severe pneumonia | Hospitalization |
|  | CVA / TIA |  |
|  | Inflammatory processes such as appendicitis, pancreatitis, cholecystitis | Hospitalization |
|  | Other, such as: exacerbation COPD or hypertensive crisis | Hospitalization |
|  | Traumatic event (with significant impact) | Hospitalization |
| **Non-major event** | Stable angina pectoris | Outpatient treatment |
|  | Mild congestive heart failure | Outpatient treatment |
|  | Mild peri(myo)carditis | Outpatient treatment |
|  | Atrial fibrillation (recurrent / paroxysmal) | Outpatient treatment |
|  | Hypertension | Outpatient treatment |
|  | Mild pneumothorax | Outpatient treatment |
|  | Mild pneumonia | Outpatient treatment |
|  | Mild respiratory problems (such as viral infections) |  |
|  | Gastric/oesophagus problems |  |
|  | Muscoloskeletal |  |
|  | Traumatic (mild trauma) | Outpatient treatment |
|  | Mental health / Panic attack / Anxiety disorder |  |

*Supplement 1. Definition of major and non-major events.*

This table specifies the subdivision between major and non-major events, and states the additional condition for some of these diagnoses in the right column.

*Abbreviations:* cerebrovascular accident (CVA), transient ischemic attack (TIA), chronic obstructive pulmonary disease (COPD).
