# Supplementary material for: Sex differences in chest pain presentation, triage assessment, and outcomes in urgent primary care: findings from the TRACE cohort study
Source: Prim Health Care Res Dev. 2025 Jul 2;26:e53. doi: 10.1017/S1463423625100182 (PMC12260727; doi:10.1017/S1463423625100182)
Supplement: Manten et al. supplementary material 2 — Manten et al. supplementary material [file S1463423625100182sup002.docx]

**Supplement 2.** Patients with an urgency code alteration, stratified by age.

**

*Figure 2. Patients with an urgency code alteration, stratified by age.*

Total number of patients represented in graph: 265.
