# Supplementary material for: Sex differences in chest pain presentation, triage assessment, and outcomes in urgent primary care: findings from the TRACE cohort study
Source: Prim Health Care Res Dev. 2025 Jul 2;26:e53. doi: 10.1017/S1463423625100182 (PMC12260727; doi:10.1017/S1463423625100182)
Supplement: Manten et al. supplementary material 3 — Manten et al. supplementary material [file S1463423625100182sup003.docx]

**Supplement 3.** Major events among women and men: specified diagnoses.

|  | | **Women**  (n=849) | **Men**  (n=621) | **p** |
| --- | --- | --- | --- | --- |
| **Major event** *(total)* | | **109 (12.8)** | **129 (20.8)** | **<0.001** |
| **Cardiovascular** | | 82 (9.7) | 102 (16.4) | <0.001 |
|  | Acute coronary syndrome | 46 (5.4) | 53 (8.5) | 0.019 |
|  | Chronic coronary syndrome *(e.g. stable angina, ischemic disease without angina)* | 5 (0.6) | 3 (0.5) | 1.00 |
|  | Atrial fibrillation | 19 (2.2) | 19 (3.1) | 0.34 |
|  | Congestive heart failure | 6 (0.7) | 7 (1.1) | 0.40 |
|  | Peri(myo)carditis | 1 (0.1) | 2 (0.3) | 0.58 |
|  | Aortic aneurysm or dissection | - | 3 (0.5) | 0.08 |
|  | Pulmonary embolism | 2 (0.2) | 8 (1.3) | 0.022 |
|  | Cerebrovascular disease *(CVA and TIA)* | 1 (0.1) | 1 (0.2) | 1.00 |
|  | Hypertension | 1 (0.1) | 1 (0.2) | 1.00 |
|  | Other cardiovascular (*tachycardias, valvular disorders, unspecified complaints)* | 3 (0.4) | 3 (0.5) | 0.70 |
| **Musculoskeletal** | | 6 (0.7) | 2 (0.3) | 0.48 |
| **Respiratory** | | 10 (1.2) | 13 (2.1) | 0.16 |
|  | Pneumonia | 5 (0.6) | 10 (1.6) | 0.05 |
|  | Pneumothorax | 1 (0.1) | 1 (0.2) | 1.00 |
|  | Chronic pulmonary diseases *(e.g. asthma, COPD)* | 2 (0.2) | 1 (0.2) | 1.00 |
|  | Respiratory complaints other | 2 (0.2) | 1 (0.2) | 100 |
| **Abdominal** | | 8 (0.9) | 9 (1.4) | 0.37 |
|  | Stomach and esophageal related complaints/diseases *(e.g. heartburn, reflux disease, nausea, indigestion, epigastric pain)* | 1 (0.1) | 1 (0.2) | 1.00 |
|  | Inflammatory & biliary *(appendicitis, chronic enteritis, pancreatitis, cholecystitis w/o cholelithiasis)* | 4 (0.5) | 5 (0.8) | 0.51 |
|  | Urological infection or calculi | 2 (0.2) | 2 (0.3) | 1.00 |
|  | Other unspecified | 1 (0.1) | 1 (0.2) | 1.00 |
| **Psychological and mental health** (*intoxication*) | | 1 (0.1) | - | 1.00 |
| **Other** *(thyroid disease, diabetes related, fever, anaphylaxis)* | | 2 (0.2) | 3 (0.5) | 0.66 |

*Supplement 2. Major events among women and men: specified final diagnoses.*

The table shows the total amount of major event for each sex and the subdivision of specific major event diagnoses. ‘Major events’ is a composite of all-cause mortality and urgent cardiovascular and non-cardiovascular conditions, occurring within 6 weeks of initial contact.

*Abbreviations:* cerebrovascular accident (CVA), transient ischemic attack (TIA), chronic obstructive pulmonary disease (COPD).
