# Supplementary material for: Sex differences in chest pain presentation, triage assessment, and outcomes in urgent primary care: findings from the TRACE cohort study
Source: Prim Health Care Res Dev. 2025 Jul 2;26:e53. doi: 10.1017/S1463423625100182 (PMC12260727; doi:10.1017/S1463423625100182)
Supplement: Manten et al. supplementary material 4 — Manten et al. supplementary material [file S1463423625100182sup004.docx]

**Supplement 4.** Patient and symptom characteristics among women and men with ACS.

|  | | | | **Women**  **With ACS** | **Men**  **With ACS** | **p** |
| --- | --- | --- | --- | --- | --- | --- |
| **Patient characteristics** | | | | 46 (5.4) | 53 (8.5) | 0.019 |
|  | Age | | | 70 (61-83) | 67 (57-75) | 0.07 |
|  | Prior CVD^*^ | | | 19 (41.3) | 21 (39.6) | 0.87 |
|  | Cardiovascular risk factors**^†^** | | | 38 (82.6) | 41 (77.4) | 0.52 |
| **Symptom characteristics** | | | | 45 (5.4) | 53 (8.6) | 0.015 |
|  | *Type of pain* | | |  |  |  |
|  |  | | Tightness/pressure | 38 (84.4) | 34 (64.2) | 0.023 |
|  |  | | Stabbing/sharp | 3 (6.7) | 3 (5.7) | 1.00 |
|  |  | | Fixed to respiration | - | 1 (1.9) | 1.00 |
|  |  | | Unknown/unclear | 4 (8.9) | 13 (24.5) | 0.042 |
|  | *Duration of chest pain* | | |  |  |  |
|  |  | <12 hours | | 38 (84.4) | 41 (77.4) | 0.38 |
|  |  | >12 hours | | 7 (15.6) | 12 (22.6) | 0.38 |
|  | *Chest pain severity (1-10 point scale)* | | | |  |  |
|  |  | | Mild (<4) | 9 (20.0) | 9 (17.0) | 0.70 |
|  |  | | Moderate (5-7) | 15 (33.3) | 23 (43.4) | 0.31 |
|  |  | | Severe (8-10) | 14 (31.1) | 14 (26.4) | 0.61 |
|  |  | | Pain has subsided / no pain | 7 (15.6) | 6 (11.3) | 0.54 |
|  | *Course of chest pain* | | |  |  |  |
|  |  | | Gradual increase in intensity | 8 (17.8) | 12 (22.6) | 0.55 |
|  |  | | Atypical and subsided | 4 (8.9) | 3 (5.7) | 0.70 |
|  |  | | Typical and subsided | - | 4 (7.5) | 0.12 |
|  |  | | Rapidly progressive | 7 (15.6) | 2 (3.8) | 0.08 |
|  | *Location on the chest* | | |  |  |  |
|  |  | | Left chest | 5 (11.1) | 7 (13.2) | 0.75 |
|  |  | | Right chest | 1 (2.2) | 2 (3.8) | 1.00 |
|  |  | | Middle (retrosternal) | 21 (46.7) | 18 (34.0) | 0.20 |
|  | *Pain radiation*^‡^ | | |  |  |  |
|  |  | | Typical cardiac radiation | 22 (48.9) | 27 (50.9) | 0.84 |
|  |  | | Atypical radiation | 2 (4.4) | 4 (7.5) | 0.68 |
|  |  | | Location not specified | 2 (4.4) | 2 (3.8) | 1.00 |
|  |  | | No radiation | 9 (20.0) | 12 (22.6) | 0.75 |
|  | *Associated symptoms*^§^ | | |  |  |  |
|  |  | | Present | 21 (46.7) | 24 (45.3) | 0.89 |
|  |  | | Subsided | 3 (6.7) | 7 (13.2) | 0.34 |
|  |  | | No associated symptoms | 11 (24.4) | 16 (30.2) | 0.53 |

*Supplement 4.* Patient and symptom characteristics among women and men with ACS.

Patient flow in the table: 46 women and 53 men were diagnosed with an ACS. Symptom characteristics could not be assessed in one of the female patients due to hemodynamic instability.

^*^ Cardiovascular disease (CVD) was defined as a history of myocardial infarction, transient ischemic attack, cerebrovascular accident, peripheral artery disease, previous percutaneous coronary intervention and/or coronary artery bypass grafting.

^†^Cardiovascular risk factors include prior CVD, a history of diabetes, hypertension, hypercholesterolemia, obesity, smoking and/or a positive family history for cardiovascular disease. For women a history with gestational hypertension or (pre)eclampsia were also considered cardiovascular risk factors.

^‡^Pain radiance was considered typical when the arm(s), shoulder(s), jaw(s) or throat were affected. All other locations of radiation were considered atypical.

^§^Associated symptoms include the presence of sweating, nausea, vomiting, pallor, anxiety, fainting and/or near collapse.

Continuous data are presented as a median and interquartile range due to a non-normal distribution. All categorical data are presented as number and percentage.
